# The Role of PSMA PET/CT in the Primary Diagnosis and Follow-Up of Prostate Cancer—A Practical Clinical Review

**DOI:** 10.3390/cancers14153638

**Published:** 2022-07-26

**Authors:** Anna Rebecca Lisney, Conrad Leitsmann, Arne Strauß, Birgit Meller, Jan Alexander Bucerius, Carsten-Oliver Sahlmann

**Affiliations:** 1 Department of Nuclear Medicine, University Medical Center Göttingen, Robert-Koch-Straße 40, 37075 Göttingen, Germany; anna.lisney@med.uni-goettingen.de (A.R.L.); birgit.meller@med.uni-goettingen.de (B.M.); jan.bucerius@med.uni-goettingen.de (J.A.B.); 2 Department of Urology, University Medical Center Göttingen, Robert-Koch-Straße 40, 37075 Göttingen, Germany; conrad.leitsmann@med.uni-goettingen.de (C.L.); astrauss@med.uni-goettingen.de (A.S.)

**Keywords:** PSMA PET/CT, prostate cancer, prostate-specific membrane antigen, PSMA, PET/CT, PET

## Abstract

**Simple Summary:**

The combination of positron emission tomography (PET)-diagnostics with ligands binding to the prostate-specific membrane antigen (PSMA) has been a diagnostic milestone in the situation of biochemical recurrence of prostate cancer and is gaining importance in primary diagnostics, providing a highly specific and sensitive diagnostic method in various clinical situations. However, the clinical application of this method requires a comprehensive knowledge of its advantages and disadvantages, potential pitfalls and influencing factors. This review aims to provide a practical clinical review of the currently available background data on PSMA PET/CT, as well as the clinical implications. Although a large amount of data already exist, a thorough analysis is complicated by study heterogeneity, showing the need for future systematic and prospective research.

**Abstract:**

The importance of PSMA PET/CT in both primary diagnostics and prostate cancer recurrence has grown steadily since its introduction more than a decade ago. Over the past years, a vast amount of data have been published on the diagnostic accuracy and the impact of PSMA PET/CT on patient management. Nevertheless, a large heterogeneity between studies has made reaching a consensus difficult; this review aims to provide a comprehensive clinical review of the available scientific literature, covering the currently known data on physiological and pathological PSMA expression, influencing factors, the differences and pitfalls of various tracers, as well as the clinical implications in initial TNM-staging and in the situation of biochemical recurrence. This review has the objective of providing a practical clinical overview of the advantages and disadvantages of the examination in various clinical situations and the body of knowledge available, as well as open questions still requiring further research.

## 1. Introduction

Prostate cancer (PCa) is the second most frequently diagnosed malignancy globally and the second leading cause of cancer-related death in men, with numbers likely to increase over the next decades, especially in developed countries, due to the aging population [1]. Since there are different treatment paths for localized, locally advanced and metastatic disease, accurate staging is essential to reduce both under- and over-treatment.

Prostate-specific membrane antigen (PSMA) as a potential diagnostic or therapeutic target has been known for a long time. As early as 1994, a murine monoclonal ^111^Indium(^111^In)-labeled IgG antibody against PSMA (^111^In-capromab pendetide; ProstaScint^®^, Cytogen Corporation, Princeton, NJ, USA) was successfully evaluated in PCa patients and approved by the FDA [2]; however, this diagnostic approach failed to gain acceptance—mainly due to the very slow kinetics/blood clearance of complete IgG antibodies resulting in a poor target/background ratio, as well as the low spatial resolution of ^111^In. Additionally, a major problem was the binding of the antibody to the intracellular domains of PSMA, and thus the visualization of primarily apoptotic tumor cells [3].

In around 2009, the possibility of synthesizing a small ^68^Gallium(^68^Ga)-labeled peptide that binds to PSMA with high affinity (a small-molecule PSMA inhibitor) opened up the possibility of widespread use of PSMA diagnostics in the context of positron-emission tomography (PET)/computerized tomography (CT) examinations [4,5]. [^68^Ga]Ga-PSMA-11, the first PSMA tracer introduced clinically in 2012 and 2013, respectively, showed a clear superiority to PET tracers such as ^18^Fluor(^18^F)-choline, which were the previous standard, by combining the high affinity to PSMA-expressing PCa cells with the high spatial resolution of PET and with a fast blood clearance, resulting in a high image contrast [6,7]. 

Since the introduction of the first PSMA tracer, several other PSMA ligands, labeled with ^68^Ga or, as another approach, with ^18^F, have been evaluated in a variety of studies for different disease settings of PCa. The possibility of labeling such tracers not only with diagnostic, but also with therapeutic radionuclides such as ^177^Lutetium(^177^Lu, a beta emitter) or ^225^Actinium(^225^Ac, an alpha emitter), allows additional use in a theranostic concept [8].

This paper aims to give an overview of the diagnostic value of PSMA PET/CT in different clinical situations—on the one hand in the primary diagnosis, and on the other hand in the recurrence situation of PCa. Additionally, the different ligands and possible influencing factors on the sensitivity and specificity of the method will be considered.

## 2. Background

### 2.1. Prostate Specific Membrane Antigen

PSMA is a type II transmembrane glycoprotein (glutamate carboxypeptidase II or N-acetyl-L-aspartyl-L-glutamate peptidase I) [9]. After binding of a small-molecule PSMA inhibitor such as [^68^Ga]Ga-PSMA-11, the PSMA-tracer-complex is internalized [10]; this internalization leads to an accumulation of the tracer in the viable tumor cells over time.

### 2.2. Physiological PSMA Expression

A strong physiological PSMA expression is detectable in normal prostate epithelium, duodenum and colon [11], as well as in sympathetic ganglia [12]; even in the proximal tubules of the kidneys, a PSMA expression could be confirmed [13]. Other tissues show variable and mostly mild expression of PSMA: normal transitional epithelium of the bladder, normal breast parenchymal elements, and weak expression in hepatocytes, testis, esophagus, stomach, small intestine, and fallopian tube epithelium [14]. Although a constant significant accumulation of the tracer in the salivary and lacrimal glands is detectable in PET/CT, this appears to be a partly nonspecific process, independent of a relevant physiological PSMA expression limited to the intercalated ducts of submandibular glandules [15,16]. An overview of the PSMA expression of different tissues is shown in Table 1.

### 2.3. Pathological PSMA Expression or Tracer Uptake

Significant pathological overexpression of PSMA is found not only in the primary PCa, but also in lymph node metastases, soft tissue metastases, and bone metastases. Furthermore, significant PSMA expression can be found in the process of neovascularization of a wide range of different tumors and their metastases as shown in Table 2.

Since the introduction of the method, uptake of the PSMA tracers has also been reported in various benign tumors (see Table 3). Unfortunately, the specificity of the PSMA uptake in the PET/CT was not systematically confirmed by immunohistochemistry in the various case reports. Frequently, there was only a histopathological or clinical confirmation (e.g., by means of magnetic resonance imaging (MRI), CT, other imaging modalities, or by means of a follow-up); this also holds true for other diseases/conditions. In general, gallium tracers are predominantly reported in the literature; fluorinated tracers may be underrepresented due to the smaller number of case reports.

PSMA uptake has also been described in various benign granulomatous or inflammatory diseases (see Table 4). The uptake mechanism is not yet fully understood; in addition to a possible accumulation of the tracer in neovascular processes, it could also be partially explained by impaired vascular permeability and increased blood flow as part of the inflammatory process, and thus by an unspecific accumulation. PSMA uptake has also been reported for various bone diseases and conditions (see Table 5).

Finally, there are several other benign or malignant conditions and diseases in which PSMA uptake has been reported (see Table 6).

### 2.4. Small-Molecule PSMA Inhibitors

Currently, the most commonly-used tracers are urea-based small-molecule inhibitors of PSMA that bind with high affinity to its enzymatic pocket [27]. Among the various tracers, a distinction can be made between diagnostic (tracers that can be labeled only with PET nuclides such as ^68^Ga or ^18^F; see Table 7) and theranostic (tracers that can be labeled with PET nuclides, such as ^68^Ga, and therapeutic nuclides, such as ^177^Lu or ^225^Ac) applications. The currently used fluorinated tracers are generally not suitable for theranostic use. Of the fluorinated PSMA tracers, only [^18^F]PSMA-1007, although not therapeutically used, is of analog structure and thus best comparable with the diagnostically and therapeutically-used PSMA-617 [28]; however, PSMA-11 and DCFPyL are already FDA-approved.

### 2.5. Gallium-PSMA vs. Fluor-PSMA

One problem with the use of ^68^Ga-labeled tracers, in general, is their short half-life, which affects the possible acquisition at later time points on the one hand, and the difficulty in producing such tracers in a central facility with higher production capacity and in transporting them to satellite facilities on the other. Another problem is the higher positron energy of gallium, which leads to poorer spatial resolution [28]. Gallium and fluorine-labeled tracers differ, among other things, in the degree of urinary excretion into the bladder. While gallium tracers have a higher urinary excretion, potentially limiting the ability to assess the prostatic compartment or surrounding pelvic lymph nodes, a delay in urinary excretion with fluorinated tracers (most notably the [^18^F]PSMA-1007) has been hailed as a distinct advantage [28]; however, a recently published paper demonstrated that within the first 10 minutes after injection of [^68^Ga]Ga-PSMA-11, the local recurrence of PCa showed significant accumulation, while the bladder did not yet show activity, thus allowing better delineation of the recurrence [29].

On the other hand, fluorinated tracers show a higher excretion via the liver and bile ducts, resulting in a potential disadvantage, especially in the assessment of visceral organs of the upper abdomen. Fluorinated tracers also tend to be inferior to gallium-labeled tracers in the assessment of bone metastases, with a more frequent occurrence of false-positive findings [30,31].

In a recently published systematic review comparing ^68^Ga and ^18^F-PSMA PET/CT, no tracer was clearly superior to the other. Theoretically, there is a possible advantage in using different tracers in different clinical situations (e.g., ^18^F-PSMA when the main focus is on local recurrence or ^68^Ga-PSMA when the focus is on retroperitoneal metastases of the upper abdomen). In practice, however, availability will probably mainly dictate the tracer used [32].

### 2.6. Pharmacokinetics and Biodistribution

**Figure 1:**
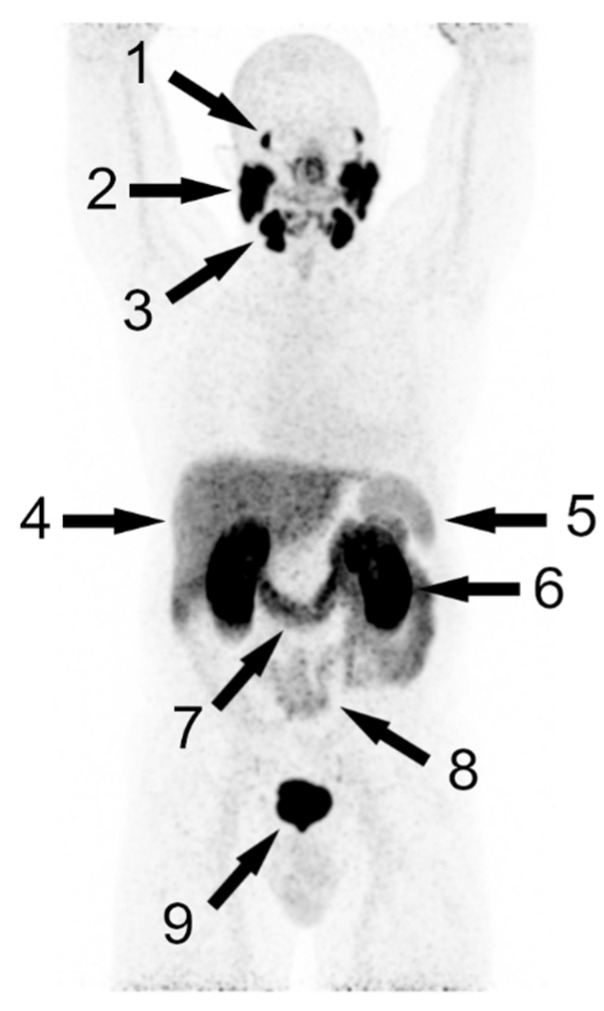
MIP of a physiological biodistribution of PSMA PET/CT in a patient after radical prostatectomy, with [^68^Ga]Ga-PSMA-11; arrows point to physiological uptake in lacrimal glands (1), parotid glands (2), submandibular glands (3), liver (4), spleen (5), kidney (6), duodenum (7), small intestines (8), and unspecific radioactivity in the bladder (9).

Although immunohistochemically, PSMA expression is detectable in a variety of tissues, a significant uptake (in descending order of intensity) in PET is found only in the kidneys, lacrimal glands, salivary glands, ureters, bladder, duodenum, and small intestines, as well as in the liver and spleen (see Table 8 and Figure 1). 

**Figure 2:**
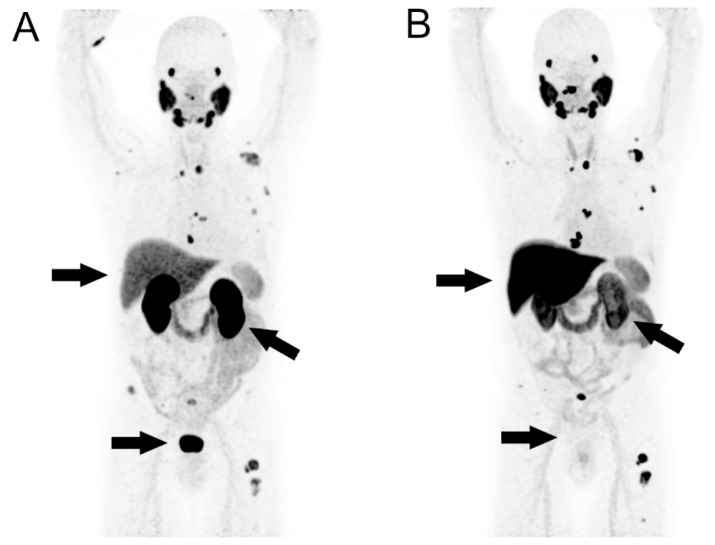
MIPs of a typical biodistribution of PSMA PET/CT studies of a patient with metastatic prostate cancer, with [^68^Ga]Ga-PSMA-11 in 2019 (**A**) and with [^18^F]-PSMA-1007 in 2018 (**B**); arrows point to significant differences in uptake.

In some cases, an uptake is detectable in the gallbladder. Certain aspects of the individual tracers differ, especially with regard to the uptake in the upper abdomen and the bladder (see Figure 2) [19,22,34]. Several normal variants have been reported, e.g., PSMA uptake in the trachea and bronchi, in mediastinal lymph nodes, in the biliary tract, in the accessory parotid gland and in sympathetic ganglia [22,34].

### 2.7. Clinical Factors and Predictors Influencing PSMA Expression or Predicting PET Positivity

Although high sensitivity and specificity were initially described for PSMA tracers, several factors are now known to potentially affect PSMA expression significantly and thus the sensitivity and specificity of PSMA PET/CT (see Table 9). Immunohistochemically, there is a direct correlation between the Gleason score and PSMA expression in the primary tumor, as well as the PET tracer uptake [35,36,37]. A complicating factor is the immunohistochemical detection of a significant heterogeneity related to PSMA expression between the primary tumor and tumor metastases, which in principle may lead to decreased sensitivity of PSMA PET/CT in metastases [38,39]. The same appears to be true for a histopathological infiltrative growth pattern of PCa, which is also associated with lower uptake in PSMA PET/CT [40,41]. Furthermore, the PSMA expression of the primary tumor does not predict the PSMA expression of the recurrence or recurrent metastases [42,43]. On the other hand, the percentage of immunohistochemically PSMA-negative tumor areas of the primary tumor seems to be predictive of PSMA PET negativity in the recurrence situation [44]. Otherwise, only the PSA level at the time of the PET scan, the PSA doubling time, and an antiandrogen therapy (ADT) were positive predictors regarding the detection rate [34,44,45,46,47]. A clear cut-off level for a specific PSA level or for a specific velocity of doubling time of the PSA with regard to an examination time has not yet been defined by consensus; however, since radiation therapy (RT) in the situation of biochemical recurrence (BCR) is recommended at PSA levels < 0.5 ng/mL, an early examination time point seems desirable.

The expression of PSMA in PCa inversely correlates with the activity of the androgen receptor. The influence of androgen deprivation therapy (ADT) on PSMA expression has been described in detail in a review paper [48]; however, the reported results of several studies are heterogeneous and results have sometimes been contradictory, making a consensus nearly impossible. Nevertheless, in principle, a distinction would be possible between a short-term effect and a long-term effect of ADT on PCa cells, independent of hormone sensitivity or a castration resistance. 

In the short term (defined as 2 to 6 weeks, depending on the study [48]), androgen receptor blockade leads to increased expression of PSMA on tumor cells, as initially shown in cell experiments. In the long term (defined as 12 to 13 weeks to 7 months, depending on the study [48]), ADT leads to decreased expression of PSMA, correlating to a decrease in serum PSA, so that the sensitivity of PET/CT potentially deteriorates in this situation, as has also been confirmed clinically [49,50]; this is true for hormone-sensitive PCa as well as castration-resistant PCa [51,52].

Chemotherapy or PSMA-ligand therapy may also affect PSMA expression. Both chemotherapy and PSMA-ligand therapy can lead to a loss of PSMA expression in metastases, giving the false impression of a response to therapy; this was shown in a therapy study, as well as in a single case report using a combination of PSMA PET/CT and an additional [^18^F]FDG PET/CT to detect dedifferentiated metastases of PCa [53,54].

### 2.8. Possible Options for Influencing PSMA Expression, Sensitivity or Specificity of PET/CT

#### 2.8.1. Dual-Time-Point Acquisition

Since the tracer is internalized after binding to the PSMA-molecule, this process can hypothetically be used to differentiate malignant from benign lesions by late acquisition in PET (dual-time-point PET/CT acquisition), thereby potentially increasing the sensitivity and specificity of the method. In several studies, this dual-time-point acquisition was evaluated not only for ^68^Ga-PSMA but also for ^18^F-PSMA in different clinical situations; however, the acquisition times differed among the studies, sometimes significantly. Nevertheless, an increase in contrast of the primary tumor, in lymph node metastases, as well as in bone metastases of the PCa of a late acquisition compared to an earlier acquisition could be shown for all different PSMA tracers. Benign findings tend to show a decrease in uptake over time, even allowing differentiation between benign prostatic hyperplasia and PCa [55,56,57,58]. Unfortunately, this does not apply to sympathetic nervous system ganglia, which are very well known to be confused with retroperitoneal lymph node metastases because of a comparable tracer uptake [12,59]; however, even if the dual-time-point acquisition does not lead to a significantly higher detection rate, it can at least facilitate the clarification of inconclusive findings [60].

#### 2.8.2. ADT Prior to PET/CT

As already described before, there is a significant increase in the expression of PSMA after antiandrogen administration in hormone-sensitive PCa, as well as in castration-resistant PCa. In principle, this effect could be used to increase the sensitivity of PET/CT by administering an antiandrogen shortly before the examination; this effect has already been described clinically in individual studies [61,62]. 

#### 2.8.3. Forced Diuresis

Especially in ^68^Ga-PSMA tracers with higher urinary excretion and therefore higher bladder activity, an additional forced diuresis (accompanied with hydration) may be useful in the recurrence situation, as it can give a better visualization and detectability of a local recurrence of PCa—even in combination with a late acquisition [63,64].

## 3. Clinical Application

### 3.1. PSMA PET/CT in Initial Staging

#### 3.1.1. Primary T-Staging

In the past decade, multiparametric MRI (mpMRI), which combines T2-weighted imaging, diffusion-weighted imaging, and a dynamic contrast-enhanced sequence, has become the key imaging modality in primary local staging of PCa. The main value of mpMRI lies in determining tumor extent and localizing index lesions for MR-targeted biopsy, which is recommended by the European Association of Urology (EAU) guidelines in combination with systematic prostate biopsies [65]. Several retrospective studies have compared the accuracy of PSMA PET/CT to mpMRI in terms of correctly identifying index lesions and evaluating tumor extent; however, results have been equivocal (see Table 10).

While one study by Sonni et al. reported no significant difference in the detection rates between either [^68^Ga]Ga-PSMA-11 PET/CT and mpMRI or the combination of the two for the detection of intraprostatic lesions, the combination of PSMA PET/CT and mpMRI slightly improved tumor localization [66]. Conversely, Berger et al. found that [^68^Ga]Ga-PSMA-11 PET/CT had better detection rates for index lesions and showed a better sensitivity in localizing index lesions than did mpMRI [67]. The authors found a relationship between the PSA level and the standard uptake value (SUV)max of detected intraprostatic lesions. Another study showed no significant difference in the detection or localization of primary PCa between ^68^Ga-PSMA PET/CT and mpMRI; however, PET/CT identified 10 of 11 lesions that were missed on MRI, and MRI identified 11 of 12 lesions missed by ^68^Ga-PSMA PET/CT [68]. Only one index lesion was missed by both imaging modalities. 

Chen et al. reported that the combination of ^68^Ga-PSMA PET/CT and mpMRI had better detection rates for clinically-significant PCa (Prostate Imaging Reporting and Data System (PI-RADS) 3) compared to mpMRI alone [69], while a study by Donato et al. reported that ^68^Ga-PSMA-PET/CT had better detection rates for multifocal and bilateral disease compared to mpMRI [70]. Regarding the assessment of extraprostatic disease, one study found that [^18^F]PSMA-1007 PET/CT may have better detection rates for seminal vesicle invasion (pT3b) compared to mpMRI (90% vs. 76%) [71], while another study found similar results for mpMRI and [^68^Ga]Ga-PSMA PET/CT or PET/MRI, but with a significantly higher inter-reader agreement for PSMA PET [72].

In summary, data are equivocal and need to be validated in prospective studies. Nevertheless, there is evidence that PSMA PET/CT and mpMRI can complement each other in primary T-staging, providing additional information on index lesions, tumor extent and safety margins. PSMA PET/MRI with an mpMRI protocol for the prostatic bed could combine the benefits of mpMRI and PSMA PET/CT in some cases, providing comprehensive information within one examination; this may be especially of value when considering to select patients for focal therapy. On the other hand, the widespread application of PSMA PET/MRI is still limited since suitable devices are currently mostly available in academic centres. As mentioned before, [^18^F]PSMA-1007 may have a higher sensitivity for intraprostatic lesions compared to ^68^Ga-PSMA, although this also needs to be further validated [73]. 

Another emerging application of PSMA PET/CT is PET-guided prostate biopsy, which has shown promising results for both [^18^F]PSMA-1007 and [^68^Ga]Ga-PSMA-11. PSMA PET/CT guided prostate biopsies may improve the diagnostic efficacy in patients with previous negative ultrasound or MRI-guided biopsies [74,75,76], especially when combined with intraoperative assessment of PSMA uptake to confirm accurate lesion sampling, as was shown by Ferraro et al. in a proof-of-concept study using [^18^F]PSMA-1007 [76].

**Table 10:** Comparison of mpMRI and PSMA PET/CT in primary T-staging.

Authors (Year)	Comparison	Number of Patients	Sensitivity PSMA PET/CT	Sensitivity mpMRI	Specificity PSMA PET/CT	Specificity mpMRI	Localization Index Tumor PSMA PET/CT (Sensitivity/Specificity)	Localization Index Tumor mpMRI (Sensitivity/Specificity)
Kalapara et al. (2020) [68]	^68^Ga-PSMA PET/CT vs. mpMRI	205	0.94	0.95	-	-	0.91/-	0.89/-
Sonni et al. (2021) [66]	^68^Ga-PSMA PET/CT vs. mpMRI vs. PSMA PET/CT + mpMRI	74	0.85	0.83	-	-	0.84/ 0.55	0.86/ 0.59
Donato et al. (2019) [70]	^68^Ga-PSMA PET/CT vs. mpMRI	58	Index lesions: 0.93 Bilateral disease: 0.42 Multifocal disease: 0.34	Index lesions: 0.90 Bilateral disease: 0.21 Multifocal disease: 0.19	-	-	-	-
Chen et al. (2019) [69]	^68^Ga-PSMA PET/CT + mpMRI vs. ^68^Ga-PSMA PET/CT or mpMRI alone	54	0.89 (95% CI: 0.79–0.96)	0.76 (95% CI: 0.64–0.86)	0.71 (95% CI: 0.49–0.87)	0.88 (95% CI: 0.68–0.97)	-	-
Berger et al. (2018) [67]	^68^Ga-PSMA PET/CT vs. mpMRI	50	Index lesions: 1.0 Secondary lesions: 0.935	Index lesions: 0.94 Secondary lesions: 0.516	-	-	0.811 (95% CI: 0.76–0.86)/ 0.846 (95% CI: 0.79–0.90)	0.648 (95% CI: 0.59–0.71)/ 0.827 (95% CI: 0.77–0.89)

All studies used histopathology obtained by radical prostatectomy as reference.

#### 3.1.2. Primary N-Staging

##### ^68^Ga-PSMA in Primary Nodal Staging

Both the EAU and the American Urology Association (AUA)/American Society of Radiation Oncology (ASTRO)/Society of Urologic Oncology (SUO) guidelines recommend further T- and N-staging in patients with intermediate and high-risk PCa (PSA > 20 ng/mL, Grade Group 4–5, T-category cT3/cT4) [65,77,78]. In primary N-staging, conventional modalities such as abdominal CT and T1-T2-weighted MRI rely on morphological features such as nodal size and shape. Lymph nodes are commonly considered suspicious if the short axis is larger than 8 mm inside and larger than 10 mm outside of the pelvis; however, there is a potential overlap in the size of metastatic and non-metastatic lymph nodes, resulting in a fairly low sensitivity of around 40% and specificity of around 80% for CT and MRI [79]. There is growing evidence that PSMA PET/CT is superior to MRI and abdominal CT in primary N-staging and various meta-analyses have been published in recent years (see Table 11). 

Wang et al. performed a meta-analysis comparing ^68^Ga-PSMA PET/CT and mpMRI for the detection of pelvic lymph node metastases using pelvic lymph node dissection (PLND) as a gold standard [80]. In conclusion, there was a trend towards a higher sensitivity and diagnostic accuracy of ^68^Ga-PSMA PET/CT. Another meta-analysis of 5 studies by Hope et al. found a high lesion-based specificity of 0.96 for ^68^Ga-PSMA PET/CT, while the pooled sensitivity was only 0.74; this is in line with another review by Luiting et al., which found a high per-node and per-patient specificity of ^68^Ga-PSMA (between 0.8 and 1.0), while the sensitivity was considerably lower with a high variability (between 0.24 and 1.0) [81].

A meta-analysis by Stabile et al. aimed to evaluate whether PSMA PET/CT can avoid the need for extended pelvic lymph node dissection (ePLND) in selected patients [82]. 27 original articles (n = 2832 patients) using different PET tracers were included in the final analysis (see Table 11). Again, this analysis found a high per-patient specificity of PSMA PET/CT of 0.95, while the overall per-patient sensitivity was relatively low with 0.58. The overall positive predictive value (PPV) and negative predictive value (NPV) were 0.79 and 0.87, respectively, and increased in patients with intermediate-risk PCa (to 0.93 and 0.96, respectively). As potential clinical implications, the authors concluded that in patients with a borderline risk of lymph node invasion (LNI) calculated by normogram and a negative PSMA PET/CT, avoiding ePLND may be considered due to the high NPV; however, in patients with a higher risk of LNI, the lower NPV means that LNI cannot be sufficiently ruled out by a negative PSMA PET/CT.

**Table 11:** Sensitivity and specificity of ^68^Ga-PSMA PET/CT for primary nodal staging.

Authors (Year)	Number of Studies Included (Patients)	Tracer	Lesion-Based Sensitivity	Lesion-Based Specificity	Patient-Based Sensitivity	Patient-Based Specificity
Stabile et al. (2022) [82]	27 (2832)	^68^Ga-PSMA, [^64^Cu]PSMA-617, [^18^F]rh-PSMA-7, [^18^F]DCFPyl, [^18^F]PSMA-1007	-	-	0.58 (95% CI: 0.50–0.66)	0.95 (95% CI: 0.93–0.97)
Tu et al. (2020) [83]	11 (904)	^68^Ga-PSMA	0.70 (95% CI: 0.49–0.85)	0.99 (95% CI: 0.96–1.00)	0.63 (95% CI: 0.46–0.78)	0.93 (95% CI: 0.88–0.96)
Luiting et al. (2020), retr. [81]	9 (696)	^68^Ga-PSMA	0.24–0.96	0.99–1.00	0.33–1.00	0.80–1.00
Wang et al. (2021) [80]	9 (640)	^68^Ga-PSMA	-	-	0.71 (95% CI: 0.48–0.86)	0.92 (95% CI: 0.88–0.95)
Hope et al. (2019) [84]	5 (266)	^68^Ga-PSMA	0.74 (95% CI: 0.51–0.89)	0.96 (95% CI: 0.85–0.99)	-	-
Luiting et al. (2020), pros. [81]	2 (63)	^68^Ga-PSMA	0.50–0.58	0.96–1.00	0.64–1.00	0.90–0.95

Retr.—retrospective; pros.—prospective.

##### ^18^F-PSMA in Primary Nodal Staging

Compared to ^68^Ga-PSMA, there have been fewer studies published on the diagnostic accuracy of ^18^F-PSMA in primary nodal staging (see Table 12). As mentioned before, because of the lower bladder activity compared to ^68^Ga-PSMA, [^18^F]PSMA-1007 may have better detection rates for (low-grade) lesions adjacent to the bladder and ureter, although these may be of limited clinical relevance [73,85].

Sprute et al. performed a retrospective, multicentre study of 96 patients who received [^18^F]PSMA-1007 PET/CT mainly for primary staging (90.6%), although 9 patients also received staging for BCR [86]. The study found a high specificity of 0.995 in the lesion-based analysis, while the sensitivity was considerably lower with 0.712. The sensitivity improved to 0.817 when only taking lymph nodes measuring more than 3 mm into account. Similar results were found in the patient-based analysis. A prospective study by Malaspina et al. compared whole-body MRI (WBMRI) and CT to [^18^F]PSMA-1007 PET/CT in 79 patients with primary intermediate or high-risk PCa [87]. Overall, the patient-based sensitivity of [^18^F]PSMA-1007 PET/CT was better, compared to conventional imaging modalities, while the specificity was similarly high. PET/CT had better inter-reader agreement compared to MRI or CT.

Kroenke et al. performed a retrospective study to evaluate the diagnostic performance of PET/CT (39 patients) or PET/MRI (19 patients) using the tracer [^18^F]rhPSMA-7, which is not currently routinely used in clinical practice, compared to conventional cross-sectional-imaging for primary N-staging in patients with high-risk PCa [88]. In this study, the PET/CT or PET/MRI images were first read purely for morphological features. After at least 4 weeks, a second read of the PET-data was performed. The results were validated by histopathology using a patient and template-based analysis. [^18^F]rhPSMA-7 PET performed significantly better than morphologic imaging alone on both patient and template-based analysis (template-based sensitivity and specificity of 0.538 and 0.969, respectively, for PET and 0.096 and 0.95 for morphologic imaging). Two patients who were initially assigned false-positives with [^18^F]rhPSMA-7 PET were found to be true-positive in clinical follow-up. 

Jansen et al. examined the diagnostic accuracy of PET/CT using the tracer [^18^F]DCFPyL (which has a significantly higher uptake in kidneys and urinary bladder compared to [^18^F]PSMA-1007 [89]) in a prospective, multicentre study including 117 patients [90]. A total of 2149 lymph nodes were resected by ePLND and the histopathology was correlated with findings from PET/CT. 17 patients had lymph node metastases in the histopathology, and of these, only 7 patients had suspicious lymph nodes in the preoperative imaging, resulting in a fairly low patient-based sensitivity of 0.412; the specificity, however, was again high (0.94). 

**Table 12:** Sensitivity and specificity of ^18^F-based PSMA tracers for primary nodal staging.

Author	Number of Patients	Tracer	Lesion-Based Sensitivity	Lesion-Based Specificity	Patient-Based Sensitivity	Patient-Based Specificity
Jansen et al. (2021) [90]	117	[^18^F]DCFPyL	-	-	0.412 (95% CI: 0.194–0.665)	0.94 (95% CI: 0.869–0.975)
Sprute et al. (2021) [86]	96 (90.6% staged before primary treatment and 9.4% following biochemical recurrence)	[^18^F]PSMA-1007	Overall: 0.712 LN > 3 mm: 0.817	Overall: 0.995 LN > 3 mm: 0.996	Overall: 0.735 LN > 3 mm: 0.859	Overall: 0.994 LN > 3 mm: 0.995
Malaspina et al. (2021) [87]	79	[^18^F]PSMA-1007	-	-	0.87 (95% CI: 0.71–0.95)	0.98 (95% CI: 0.89–1.00)
Kroenke et al. (2020) [88]	58	[^18^F]rhPSMA-7 (PET/CT or PET/MRI)	0.538 (95% CI: 0.413–0.660) (template based)	0.969 (95% CI: 0.914–0.989) (template based)	0.722 (95% CI: 0.465–0.903)	0.925 (95% CI: 0.796–0.984)

LN, lymph nodes.

##### Summary—PSMA PET/CT in Primary Nodal Staging

In light of the data published in recent years, PSMA PET/CT has been recommended by the EAU as being more appropriate in N-staging compared to MRI, abdominal contrast-enhanced CT or choline PET/CT, and it may be considered as an alternative to conventional imaging in the initial staging of high-risk PCa patients, but to date no outcome data exist to inform subsequent management [65]. All included studies show a specificity of more than 90% in both the lesion and the patient-based analysis. On the other hand, despite being much better than conventional imaging, the sensitivity still appears to show scope for improvement, meaning that foregoing the gold standard PLND on the basis of PET results may currently only be considered in very select patient groups. Additionally, certain tracer-specific pitfalls should be considered [32]. Moreover, as with conventional imaging modalities, there is a size relationship with regard to lymph node metastases that are missed by PET, although this seems to be considerably lower (around 5 mm) [88,91], and the sensitivity of PSMA PET/CT increases substantially when only lymph nodes measuring more than 3 mm are taken into account [86].

#### 3.1.3. Primary M-Staging

The most common sites of PCa metastases are bone (84%), distant lymph nodes (10.6%), liver (10.2%) and thorax (9.1%) [92]. According to European and American guidelines, symptomatic patients, patients with high-risk PCa, as well as patients with unfavourable intermediate-risk PCa should be staged using cross-sectional body imaging with CT or MRI and ^99m^technetium bone scan (BS) [65,93]. On the other hand, imaging overuse in low-risk patients should be avoided [94,95]. According to the EAU guideline, this traditional workup can be replaced by the more sensitive PSMA PET/CT in the initial staging of high-risk PCa patients [65]. Systematic high-level data on the diagnostic accuracy of PSMA PET/CT for the detection of visceral metastases is lacking, so the following section will focus mainly on bone metastases. BS with single photon emission computed tomography (SPECT) has a pooled sensitivity and specificity for bone metastases of 79% (95% CI: 73–83%) and 82% (95% CI: 78–85%), respectively [96]. The specificity and diagnostic confidence can be improved by the application of SPECT/CT [97,98,99] and the main advantages lie in the wide availability and cost-effectiveness.

While the incidence of bone metastases correlates with the PSA level, bone metastases also occur in patients with serum PSA level < 5 ng/mL [100]. A retrospective analysis found PSMA-positive bone lesions in 12 of 93 patients (13%) who underwent initial staging, 40 of 225 (20%) of patients with BCR and in 49 of 70 patients (70%) who underwent restaging of M1 disease [100]. In patients with PSA level < 5 ng/mL, 17.6% of patients had PSMA-positive bone lesions with similar numbers in all three patient groups; this indicates a potential benefit of PSMA PET/CT in patients with lower PSA levels, in whom the metastatic disease may otherwise be missed.

A multicentre, randomised prospective study by Hofman et al. compared the diagnostic accuracy for pelvic nodal and distant metastases of CT and bone scan to [^68^Ga]Ga-PSMA-11 PET/CT in 300 high-risk PCa patients [101]. The study found a superior diagnostic accuracy for PSMA PET/CT for both the detection of pelvic nodal disease (AUC 0.91 vs. 0.59) and distant metastases (AUC 0.95 vs. 0.74). The finding was attributed to a high tumor-to-background contrast and the specificity of the tracer; moreover, first-line imaging with conventional imaging had a higher radiation exposure compared to PSMA PET/CT (19.2 mSv vs. 8.4 mSv).

The superior sensitivity and specificity of PSMA PET/CT compared to BS is further supported by a systematic review by Zacho et al. [102]. The largest study by Pyka et al. included 37 patients for initial staging and reported a patient-based sensitivity of 1.0 and specificity of 0.913–1.0 for ^68^Ga-PSMA PET/CT (compared to 0.571–0.714 and 0.652–0.957 for BS, respectively) [103]. PSMA PET/CT also had the highest per-patient sensitivity and specificity (0.97 and 1.00, respectively) in another systematic review comparing PSMA PET/CT, choline PET/CT, sodium fluoride (NaF) PET/CT, MRI and BS in both initial staging and BCR [104].

In summary, PSMA PET/CT appears to have a superior sensitivity and specificity compared to the traditional workup, although further high-level data are needed. As histopathological confirmation is often lacking, false-positive findings are difficult to exclude. It should also be noted that prospective studies on treatment and outcome are still based on the definition of metastatic disease by CT scan and bone scintigraphy. The subsequent management of patients with metastases seen only on the more sensitive PSMA PET/CT (e.g., systemic therapy vs. aggressive local and metastasis-directed therapy) is therefore still unclear.

### 3.2. Biochemical Failure

Between 5% and 20% of patients with PCa present with a persistent PSA > 0.1 ng/mL following radical prostatectomy, which may result from remaining local disease or benign prostate tissue as well as pre-existing metastatic disease. Biochemical persistence (BCP) has been shown to be a negative prognostic factor and is associated with higher tumor stages, higher Gleason scores and positive surgical margins [105,106,107,108]. Conventional imaging has a low sensitivity in patients with a PSA < 2 ng/mL, and PSMA PET/CT can improve detection rates for further treatment planning (e.g., salvage RT, metastasis-directed therapy or systemic therapy) [109]. The positivity rates of PSMA PET/CT correlate with the PSA level and range from 33% in men with post-RP PSA between 0–0.19 ng/mL to 97% in men with PSA > 2 ng/mL [110,111,112,113,114,115].

A retrospective study by Meijer et al. included 150 hormone-naïve patients with BCP after robot-assisted radical prostatectomy who were staged with either [^18^F]DCFPyL PET/CT or [^68^Ga]Ga-PSMA-11 PET/CT [116]. 101 patients had visible lesions with increased PSMA expression, of these 62 patients had lesions limited to the pelvic area, 13 patients had lesions only outside and 26 had lesions both inside and outside of the pelvic area. 89 patients had PSMA-avid sites outside the prostatic fossa and 39 patients had evidence of distant metastatic disease. This study indicates that a significant number of patients with biochemical persistence have evidence of extrapelvic disease or distant metastases, highlighting the value of PSMA PET/CT for determining the correct course of treatment in this situation. In a further retrospective study by Farolfi et al., [^68^Ga]Ga-PSMA-11 PET/CT localised PCa in 130 of 191 high-risk PCa patients (68%) with a positive predictive value of 91% [117]. The most frequently affected nodal regions were the obturator and the presacral/mesorectal region. Especially the mesorectum presents a possible blind spot in the traditional workup of PCa because of the low diagnostic accuracy of conventional imaging for lymph nodes measuring less than 10 mm in diameter and since this region is not included in standard PLND [118]; however, accurate localization of lymph node metastases is essential for further treatment planning and optimization of the radiation field. Hijazi et al. reported the detection of mesorectal nodal metastases on ^68^Ga-PSMA PET/CT in 15.8% of patients with high-risk PCa or BCR.

Schmidt-Hegemann et al. studied 129 patients, of these 52% with BCP, who received PSMA PET/CT-based radiotherapy [119]. The study found this treatment to be effective with significant PSA response in both patients with BCR and biochemical persistence. 

### 3.3. PSMA-PET in the Situation of Biochemical Recurrence

#### 3.3.1. Local Recurrence

After initial curative treatment (radical prostatectomy or RT) around 15–40% of patients develop a BCR (defined as PSA ≥ 0.2 ng/mL following radical prostatectomy or a PSA rise of 2.0 ng/mL or more above the nadir following RT) [120]. The risk of local recurrence (LR) following radical prostatectomy is associated with positive surgical margins and locally advanced disease and the main treatment is salvage RT [65,121,122]. MRI may detect local recurrence, however detection rates at PSA < 0.5 ng/mL are low [123]. The sensitivity may be improved by endorectal coil (e-coil) MRI, although further studies are necessary [124]. Data on the additional value of PSMA PET/CT for the detection of local recurrence are relatively sparse. In a meta-analysis by Perera et al., overall estimates of positivity of ^68^Ga-PSMA PET/CT in the prostate bed was 28% [125]. ^68^Ga-PSMA avidity was significantly higher in the radiotherapy cohort than in the prostatectomy cohort (52% vs. 22%). Assessing only the prostatic bed, Freitag et al. retrospectively analysed 119 patients who received [^68^Ga]Ga-PSMA-11 PET/CT (1 h p.i.) and [^68^Ga]Ga-PSMA-11 PET/MRI (3 h p.i.) including an mpMRI protocol for the prostatic bed [126]. mpMRI detected significantly more intraprostatic lesions compared to the PET-data of both PET/CT and PET/MRI (18 patients vs. 9 patients, *p* = 0.004). There was no [^68^Ga]Ga-PSMA-11 PET-positive/MRI-negative lesion. The detection of LR using the PET-component was significantly influenced by proximity to the bladder. 

#### 3.3.2. Secondary N- and M-Staging

An accurate restaging in the situation of BCR is essential for determining the correct course of treatment, i.e., salvage RT extended to the pelvic lymph nodes, metastasis-targeted and/or systemic treatment, or new options such as salvage lymph node dissection. For many years, standard imaging to assess metastatic disease burden in this situation included CT or MRI and bone scintigraphy, albeit with low diagnostic accuracy. The EAU guideline recommends performing a PSMA PET/CT at early BCR (PSA > 0.2 ng/mL) if the results will influence subsequent treatment decisions [65]. PSMA PET/CT has a higher sensitivity, especially in patients with lower PSA levels, compared to morphologic imaging or choline or fluciclovine PET/CT with positivity rates increasing with higher PSA levels [127,128,129,130,131]. At PSA < 0.2 ng/mL, positivity of ^68^Ga-PSMA PET/CT has been shown to be 33%, increasing to 97% at PSA > 2 ng/mL [125]. Similar results were found by another meta-analysis [132]. For ^18^F-labeled tracers, a meta-analysis by Treglia et al. reported a pooled detection rate of 49% for PSA < 0.5 ng/mL, 86% for PSA > 0.5 ng/mL and 92% for PSA ≥ 2 ng/mL [133]. By comparison, a small prospective study found detection rates for ^18^F-fluoromethylcholine PET/CT of 12.5% for PSA < 0.5 ng/mL and 63% for PSA > 2.0 ng/mL, which were significantly lower than those of ^68^Ga-PSMA PET/CT [134].

In a review by Perera et al., overall estimates of positivity of ^68^Ga-PSMA PET/CT in the restaging setting was 38% in pelvic lymph nodes, 13% in extrapelvic lymph nodes, 22% in bone and 5% in distant viscera (see Table 13) [125]. With respect to the accuracy of lymph node detection in BCR, most data are from studies using PSMA PET/CT before salvage lymphadenectomy. Kimura et al. performed a systematic review and meta-analysis of the performance of [^68^Ga]Ga-PSMA-11 PET/CT for detecting lymph node metastases in patients with recurrent PCa [135]. The analysis included 14 studies (462 patients). The authors found a pooled sensitivity and specificity in the lesion-based analysis of 0.84 (95% CI: 0.61–0.95) and 0.97 (95% CI: 0.95–0.99), respectively. Pooled sensitivity and specificity in the field-based analysis were 0.82 (95% CI: 0.72–0.89) and 0.95 (95% CI: 0.70–0.99). Included in the analysis was a retrospective study by Rauscher et al. which compared [^68^Ga]Ga-PSMA-11 to morphologic imaging with CT or MRI for the assessment of lymph node metastases (LNM) in 41 patients with recurrent PCa [127]. The study found a high specificity of PSMA PET/CT and morphologic imaging of 97.3% and 99.1%, respectively; however, PSMA PET/CT had significantly superior detection rates, detecting 53 of 68 histopathologically proven LNM (77.9%), whereas morphological imaging only detected 18 (26.9%). A meta-analysis by Pozdnyakov assessed the impact of PSMA PET/CT on patient management and clinical outcomes in the situation of BCR. PSMA PET/CT was positive in 68.2% of patients and resulted in a change in management in 56.4% (see Table 13) [136]. The pooled rate of biochemical recurrence-free survival was 60.2% at a median follow-up of 20 months; this is further supported by a meta-analysis by Wondergem et al., which also assessed the clinical impact of PSMA PET/CT; this study found that PSMA PET/CT resulted in a change in patient management in 45% of patients (*n* = 861/1899) [137]. A subgroup analysis revealed that in 50%, the treatment approach was changed from systemic treatment or surveillance to a targeted approach, while a change from a targeted to a non-targeted approach was found in 17% of patients. 

Overall, it can be concluded that PSMA PET/CT has significantly improved diagnostic imaging in the situation of BCR, often leading to a change in treatment course. Nevertheless, further data with a homogenous patient population would be valuable and facilitate a thorough analysis.

##### Metastasis-Directed Therapy Based on Results from PET/CT

Interest in metastasis-directed therapy (MDT) has grown in recent years as this treatment approach can potentially prevent additional metastatic spread, improve survival and postpone the need for systemic therapy, improving the patient’s quality of life [138]. A retrospective study by Steuber et al. found that MDT based on results of either ^11^C-choline or ^18^F-fluorethylecholine PET/CT was associated with improved cancer-specific survival compared to standard of care in 2079 patients with BCR [139]. Ost et al. performed a multicentre, randomized study of 62 patients who had three or fewer extracranial metastatic lesions on choline PET/CT and received either MDT or underwent surveillance [138]. The study found a longer ADT-free survival with MDT than surveillance alone. Only very few studies have been published on the results of MDT based on results from PSMA PET/CT [140]. Mazzola et al. compared the impact of ^18^F-choline and ^68^Ga-PSMA PET/CT-guided MDT with stereotactic body radiotherapy (SBRT) in patients with castration-sensitive oligorecurrent PCa [141]. The study found that PSMA PET/CT-guided SBRT showed better results compared to the ^18^F-choline PET/CT cohort with a higher rate of ADT-free patients, indicating that further research in this direction would be valuable. Currently, a randomized prospective phase III trial is investigating salvage RT based on [^68^Ga]Ga-PSMA-11 PET/CT compared to standard salvage RT [142].

#### 3.3.3. How to Deal with Negative PSMA PET/CT despite Rising PSA?

An important question is the frequency of negative PSMA PET/CT examinations in patients with BCR, especially with regard to further diagnostic and therapeutic procedures. In a meta-analysis, the overall detection rate of PSMA PET/CT in the BCR setting was 74.1%, regardless of the tracer used, corresponding to a negative rate of 25.9% [47]. In terms of such a negative rate, a working group—albeit with only a small collective of 29 patients—investigated whether a repeated PSMA PET/CT after a negative scan in patients with a BCR of PCa is useful. The authors reported a positive result in 41.3% of repeated PET examinations during follow-up, resulting in a change of therapy in 20.6% of patients. While the sensitivity of the first examination correlated with the PSA level, no independent predictive parameter was found for the repeated examination [143]. The authors discussed possible tumor de-differentiation as a cause for a negative PET/CT. Such de-differentiation can in principle be detected by [^18^F]FDG PET/CT. Here, another work demonstrated that patients with a negative PSMA PET/CT in the BCR setting may benefit from an examination with [^18^F]FDG. Of 72 patients with a negative PSMA PET/CT, positive PET findings could still be detected in 12 patients (16.7%), and these patients had higher Gleason scores and higher PSA levels than the [^18^F]FDG-negative patients [144]. Despite those additional positive findings in a repeated PSMA PET/CT examination and the possible detection of de-differentiated tumors by [^18^F]FDG PET/CT, a considerable group of patients remains in whom tumor manifestations can still not be found in the imaging procedures. 

In the case of a negative PSMA PET/CT, choline tracers are an important diagnostic alternative, even if choline tracers seem to be inferior to PSMA-based tracers, especially at low PSA values, as shown in a meta-analysis [145]; however, there is currently no systematic literature investigating this constellation. Other PET tracers potentially suitable for diagnostics of BCR in PCa, such as ^68^Ga-FAPI (fibroblast activation protein inhibitor), have not yet entered routine use and are still being evaluated in individual studies.

However, a negative PSMA PET/CT in patients with a BCR of PCa appears to be a positive predictor of a high response to salvage radiotherapy of the prostate compartment or in biochemical failure [146]. A small study of patients with a negative PSMA PET/CT found a treatment response in 85% of patients who received SRT (23 of 27 patients), while 65% of patients not treated showed a further increase in PSA (22 of 34 patients) [146]. Ultimately, however, further studies need to investigate potential diagnostic pathways in these patients.

### 3.4. PSMA PET/CT in Therapy

#### 3.4.1. Optimal Time Point for PSMA PET/CT

An important question that arises from the fact that ADT influences PSMA expression in PCa (see Section 2.7) is the question of the optimal time point for PSMA PET/CT diagnostics. As already mentioned, short-term effects can be distinguished from long-term effects:

1. Short-term ADT in hormone-sensitive PCa: In a small study, Emmett et al. were able to show that in hormone-sensitive patients, a significant reduction of PSMA uptake in the PET occurs very early (within 9 days), so that an examination should ideally take place before the start of the therapy [147]; this was also recommended as part of a consensus statement based on the same study [148]. In individual cases, however, an initial increase in uptake by day 9 can occur even in hormone-sensitive patients, which is in line with the contradictory data mentioned above. It is possible that these cases represent initial hormone-resistant PCa tumor clones [147].
2. Long-term ADT in hormone-sensitive PCa: If ADT has already been started in hormone-sensitive PCa, the same consensus statement does not recommend PSMA PET/CT within the first three months [148].
3. Short-term ADT in castration-resistant PCa: In patients with castration-resistant PCa, Emmet et al. were also able to show that the tumors experience a significant change in PSMA uptake in PET/CT. Within 9 days—with a plateau of up to 28 days after the start of ADT—the uptake increased, so that the optimal time seems to lie within this period if the potentially increased sensitivity is used for initial diagnostics [147].
4. Long-term ADT in castration-resistant PCa: In order to detect a therapeutic effect of ADT in castration-resistant PCa, a further examination should be performed in these patients after three months at the earliest [148].

There is no consensus recommendation for castration-resistant PCa regarding the optimal timing of PSMA PET/CT. A major problem is that apart from the cited single study, which only investigated the situation under ADT, no further systematic data are available so far. In addition, it must be mentioned that the consensus recommendation regarding the timing of PSMA PET/CT under systemic therapy in hormone-sensitive PCa is based on this single study only and thus the recommendation is derived from data under ADT. Because ADT is essentially used as a systemic therapy, a generalization is understandable in principle from basic theoretical considerations; however, there are currently no systematic data for the optimal timing of PSMA PET/CT under chemotherapy or radionuclide therapy (^223^Radium (^223^Ra), ^177^Lu-PSMA, or ^225^Ac-PSMA).

#### 3.4.2. PSMA PET/CT in Monitoring and Response Assessment of Therapy

In addition to the use of PSMA PET/CT in primary and recurrence diagnostics, it can also be used in monitoring PCa therapy and assessment of response. In a recent systematic review, Alongi et al. examined the use of PSMA and choline PET/CT to evaluate response and survival of the treatment of PCa with radiotherapy, chemotherapy, and ADT [149]. Articles assessing the response to immunotherapy or radioligand therapy (RLT) were not included. From radionuclide therapies, only the use of PET/CT in therapy with ^223^Ra was considered. Twenty-three papers for PSMA PET/CT were included. [^68^Ga]Ga-PSMA-11 was used as a tracer in all articles, except for one paper where [^18^F]DCFPyL was used. 

Here—to highlight only a few points—it could be shown that PET-based salvage radiotherapy in the recurrence situation provides similarly good results as ADT. Another promising result is excellent local control in oligometastatic PCa by PSMA-PET-based SBRT or metastasis-directed therapy, which also showed improved overall survival. For the use of PSMA PET in the context of ADT, in addition to the points mentioned in Section 3.4.1., it could be shown that the SUV values after ADT treatment were associated with the outcome and the change in the uptake pattern of PSMA PET/CT after therapy was associated with the time of progression and overall survival. Regarding the use of PSMA PET in a chemotherapy setting, it possibly showed superiority over conventional imaging, with baseline PSMA tumor volume being a possible predictor for first-line therapy with docetaxel. Finally, PSMA PET/CT in combination with bone scan may be a useful predicting response to ^223^Ra therapy.

A fundamental problem that becomes apparent in this review is that in many articles, a qualitative PET evaluation was performed without the application of standardized response criteria. Here, attempts are being made to establish appropriate criteria for standardized evaluation of PSMA PET/CT, such as the PSMA-PET-specific “The PSMA PET Progression Criteria” proposed by Fanti et al., or the RECIP 1.0 criteria proposed by Gafita et al., which have already been successfully evaluated clinically in RLT and compared to the previously established non-PSMA-PET-specific response criteria [150,151,152]. 

Regarding RLT with ^177^Lu-PSMA or ^225^Ac-PSMA, PSMA PET/CT is a major part of the theranostic concept, not only for assessment of therapeutic response, but also for the initial selection of patients who are suitable for RLT [153]. In a study, Grubmüller et al. retrospectively investigated the use of PET/CT with [^68^Ga]Ga-PSMA-11 in ^177^Lu-PSMA therapy and could show that the total tumor volume (TTV) in PET seems to be a reliable parameter for response assessment, especially since the change of TTV under therapy was also linked to overall survival [154]. Rosar et al. could also confirm these data [155].

The very heterogeneous study situation for PSMA PET/CT in PCa therapy reflects the wide range of different therapeutic options; however, to date the literature is insufficient to address these issues adequately. Systematic studies using standardized findings are urgently needed here.

## 4. Conclusions

The introduction of PSMA PET/CT was a diagnostic milestone in the situation of biochemical recurrence of PCa and is gaining importance in primary PCa diagnostics. For the first time, highly sensitive and highly specific diagnostics could be offered in various clinical situations. Nevertheless, the application of this method requires a comprehensive knowledge of the various influencing factors that can affect the diagnostic outcome. Overall, the study situation is very heterogeneous, especially regarding the distinction between different clinical situations; moreover, the large variety of tracers, different acquisition times and the lack of a gold standard have often hindered reaching a consensus. A comprehensive standardization of clinical studies would be desirable and might be advanced by the recent FDA-approval of several PSMA tracers. Future studies should aim to fully characterize the patient collective and clearly distinguish between different clinical stages. Additionally, further research of factors intentionally or unintentionally influencing the sensitivity and specificity of the method would be valuable to improve the diagnostic outcome. Here, further studies should not only systematically evaluate the role of PSMA PET/CT in primary and recurrence diagnostics, but also address the use of PSMA PET/CT in the evaluation and outcome monitoring of systemic therapies, with particular reference to RLT, as a promising new therapeutic option.

## Figures and Tables

**Table 1:** Immunohistochemically proven physiological PSMA expression in different tissues (independent of the immunohistochemically measured strength of PSMA expression).

Physiological PSMA Expression
Submandibular glands
Normal prostate epithelium
Duodenum
Colon
Proximal tubules of the kidney
Sympathetic ganglia
Normal transitional epithelium of the bladder
Normal breast parenchymal elements
Hepatocytes
Testis
Esophagus
Stomach
Small intestine
Fallopian tube epithelium

**Table 2:** Immunohistochemically proven pathological PSMA expression in different malign tumors (independent of the immunohistochemically-measured strength of PSMA expression).

Pathological PSMA Expression (Primary Tumor and Metastases)
Prostate carcinoma
Renal carcinoma *
Bladder carcinoma *
Brain tumors *
Thyroid tumors *
Hepatocellular carcinoma *
Lung carcinoma *
Squamous cell carcinomas of oral cavity *
Adenoid cystic carcinoma *
Salivary duct carcinoma *
Adrenocortical carcinoma *
Gynecologic malignancies *
High-grade sarcomas *
Pancreatic carcinoma *
Colorectal carcinoma *
Gastric carcinoma *
Intestinal adenocarcinoma (*)

Table adapted from Refs. [11,14,17,18,19,20]. * PSMA expression only in the neovascularization; (*) immunohistochemically location of PSMA expression not reported.

**Table 3:** Immunohistochemically, histologically or clinically (MRI, CT, scintigraphy, etc.) proven pathological tracer uptake in different benign tumors (independent of the strength of PSMA uptake).

Pathological Tracer Uptake (Benign Tumors)
Elastofibroma dorsi
Dermatofibroma
Acrochordon
Fibromatosis desmoid tumor
Intramuscular myxoma
Pseudoangiomatous stromal hyperplasia of the breast
Thymoma
Thyroid adenoma
Parathyroid adenoma
Adrenal adenoma
Meningioma
Schwannoma
Peripheral nerve sheath tumors
Neurofibroma
Hemangioma
Angiolipoma
Hemangiopericytoma

Table adapted from Ref. [19].

**Table 4:** Immunohistochemically, histologically or clinically (MRI, CT, scintigraphy, etc.) proven pathological tracer uptake in different granulomatous or inflammatory diseases (independent of the strength of PSMA uptake).

Pathological Tracer Uptake (Granulomatous or Inflammatory Diseases)
Pulmonary sarcoidosis
Wegener’s granulomatosis
Bronchiectasis
Anthracosilicosis
Berylliosis
Pulmonary histoplasmosis
Tuberculosis
Asbestosis
Perianal fistula
Renal abscess
Post-operative inflammatory processes
Crohn’s disease

Table adapted from Refs. [19,21,22,23].

**Table 5:** Immunohistochemically, histologically or clinically (MRI, CT, scintigraphy, etc.) proven pathological tracer uptake in different benign bone diseases (independent of the strength of PSMA uptake).

Pathological Tracer Uptake (Bone Diseases)
Fracture *
Osteophyte
Osteoarthritis
Paget’s disease +
Osteomyelitis
Fibrous dysplasia
Hemangioma

Table adapted from Refs. [19,21,22]. All conditions have been reported in [^68^Ga]Ga-PSMA-11 PET/CT; * pathological uptake additionally reported in [^18^F]PSMA-1007 PET/CT; + pathological uptake additionally reported as in [^18^F]DCFPyL PET/CT.

**Table 6:** Immunohistochemically, histologically or clinically (MRI, CT, scintigraphy, etc.) proven pathological tracer uptake in various diseases/findings (independent of the strength of PSMA uptake).

Pathological Tracer Uptake (Various Diseases/Findings)
Lymphoma
Testicular tumors
Thymic carcinoma
Polycythemia vera (diffuse bone marrow uptake)
Atelectasis
Amyloidosis of the seminal vesicles
Gynecomastia
Barrett’s esophagus

Table adapted from Refs. [14,19,21,22,24,25,26].

**Table 7:** PSMA-ligands, radionuclides and potential application.

Tracers	Nuclides	Class
PSMA-11	^68^Ga	Diagnostics
PSMA-617	^68^Ga, ^177^Lu, ^225^Ac	Theranostics
PSMA-I&T	^68^Ga, ^177^Lu, ^225^Ac	Theranostics
DCFPyL	^18^F	Diagnostics
DCFBC	^18^F	Diagnostics
PSMA-1007	^18^F	Diagnostics

Table adapted from Ref. [9].

**Table 8:** Physiological PSMA uptake in different Organs with [^68^Ga]Ga-PSMA-11 PET/CT adapted from Hofman et al. [33].

Organ	Uptake
Kidney	+++
Lacrimal glands	+++
Parotid glands	+++
Submandibular glands	+++
Duodenum	++
Liver	+
Spleen	+
Small Intestine	+

+, ++, +++ = moderate-, high-, and very high–intensity uptake, respectively.

**Table 9:** Possible factors influencing PSMA expression; possible predictors of PSMA PET positivity.

**Factor**	**Effect**
Intrinsic (primary tumor, metastases)	Heterogeneity of PSMA expression
PSMA therapy	Loss of PSMA expression
Chemotherapy	Loss of PSMA expression
ADT—short term	Increase of PSMA expression
ADT—long term	Decrease of PSMA expression
Gleason score	PSMA expression primary tumor
**Predictors**	**Prediction**
Primary tumor % PSMA negativity	PSMA PET negativity
Primary tumor growth pattern	PSMA PET uptake
PSA-value	PSMA PET positivity
PSA doubling time	PSMA PET positivity

**Table 13:** Meta-analyses of PSMA PET/CT in the situation of biochemical recurrence.

Authors (Year)	Number of Studies Included (Patients)	Tracers	Overall Positivity	Change in Patient Management
Tan et al. (2019) [132]	43 (5113)	[^18^F]DCFPyL, [^18^F]DCFBC, [^68^Ga]Ga-PSMA-11, [^18^F]PSMA-1007, [^68^Ga]Ga-PSMA I&T	70.2%	-
Pozdnyakov et al. (2022) [136]	34 (3680)	^68^Ga-PSMA and ^18^F-labeled PSMA tracers	68.2%	56.4% (95% CI: 48.0–63.9%)
Perera et al. (2020) [125]	30 (4476)	^68^Ga-PSMA tracers	28% prostate bed 38% pelvic lymph nodes 13% extrapelvic lymph nodes 22% bone 5% distant viscera	-
Wondergem et al. (2020) [137]	16 (1899)	[^68^Ga]Ga-PSMA-11, [^18^F]DCFPyL, [^18^F]PSMA-1007, [^68^Ga]Ga-THP-PSMA	-	45%
Treglia et al. (2019) [133]	6 (645)	[^18^F]PSMA-1007, [^18^F]DCFPyL, [^18^F]DCFBC	81% (95% CI: 71–88%)	-
